# (*m*, *n*)-mer—a simple statistical feature for sequence classification

**DOI:** 10.1093/bioadv/vbad088

**Published:** 2023-07-11

**Authors:** Amanda Araújo Serrão de Andrade, Marco Grivet, Otávio Brustolini, Ana Tereza Ribeiro Vasconcelos

**Affiliations:** Bioinformatics Laboratory (LABINFO), National Laboratory for Scientific Computing, Av. Getulio Vargas, 333—Quitandinha, 25651-076, Rio de Janeiro, Brazil; Pontifícia Universidade Católica do Rio de Janeiro, Rua Marquês de São Vicente 225, Gávea, 22451-900, Rio de Janeiro, Brazil; Bioinformatics Laboratory (LABINFO), National Laboratory for Scientific Computing, Av. Getulio Vargas, 333—Quitandinha, 25651-076, Rio de Janeiro, Brazil; Bioinformatics Laboratory (LABINFO), National Laboratory for Scientific Computing, Av. Getulio Vargas, 333—Quitandinha, 25651-076, Rio de Janeiro, Brazil

## Abstract

**Summary:**

The (*m*, *n*)-mer is a simple alternative classification feature based on conditional probability distributions. In this application note, we compared *k*-mer and (*m*, *n*)-mer frequency features in 11 distinct datasets used for binary, multiclass and clustering classifications. Our findings show that the (*m*, *n*)-mer frequency features are related to the highest performance metrics and often statistically outperformed the *k*-mers. Here, the (*m*, *n*)-mer frequencies improved performance for classifying smaller sequence lengths (as short as 300 bp) and yielded higher metrics when using short values of *k* (ranging from 2 to 4). Therefore, we present the (*m*, *n*)-mers frequencies to the scientific community as a feature that seems to be quite effective in identifying complex discriminatory patterns and classifying polyphyletic sequence groups.

**Availability and implementation:**

The (*m*, *n*)-mer algorithm is released as an R package within the CRAN project (https://cran.r-project.org/web/packages/mnmer) and is also available at https://github.com/labinfo-lncc/mnmer.

**Supplementary information:**

Supplementary data are available at *Bioinformatics Advances* online.

## 1 Introduction

Alignment-free feature extraction methods are well-established in comparing genomes that do not share an alignable set of common genes (Zielezinski *et al.*, 2019). These methods assess sequence similarity without resorting to sequence alignment and overcome the limitations of well-established alignment-based methods (Ren *et al.*, 2018; Zielezinski *et al.*, 2019). These alignment-free features are evaluated based on how frequently they appear in a sequence, rather than just their position (Ren *et al.*, 2018). Given this characteristic, alignment-free methods can compare low conservation sequences, even in the presence of distinct genome organizations, shuffling or recombination (Vinga, 2014). This area is under active research and new alignment-free feature extraction methods are being developed to improve sequence comparison and classification (Zielezinski *et al.*, 2019).

Among the available alignment-free methods, the *k*-mer-based classification technique is widely used and presents high discriminatory capability in several biological problems (Ren *et al.*, 2018). A *k*-mer denotes a contiguous sequence of nucleotides with a length of *k*, which is obtained from diverse biological data sources. It has been used for classification purposes, embedded in numerous frameworks such as machine learning (ML) (Alam and Chowdhury, 2020; Zhang *et al.*, 2019) and deep learning (Ren *et al.*, 2020) algorithms. Choosing an optimal value for the parameter *k* still represents a complex issue. Increasing the *k*-mer size requires larger sequences that may enhance classification performance at a high computational cost. As a result, there is an ongoing need to develop alignment-free methods with high classification performance and low computational effort (Lebatteux et al., 2019), especially when big polyphyletic databases are involved.

Classification methods are commonly based on numeric features taken from short biological sequences, and their proper selection greatly impacts their discriminatory power. Alignment-free features employed in sequence classification should be: (i) easy to obtain, (ii) capable of coping with large amounts of data and (iii) capable of exhibiting low processing time. Additionally, these features cannot rely on *a priori* assumptions about sequence evolution (Lebatteux *et al.*, 2019; Zielezinski *et al.*, 2019).

Here, we present a simple alternative alignment-free feature extraction method named (*m*, *n*)-mer and investigate it for the specific problem of classification. The (*m*, *n*)-mer is a *k*-mer variant feature that expresses conditional probabilities instead of unconditional ones. When *m* + *n* = *k*, this new feature has an identical size and range to this *k*-mer. In this study, we assessed (*m*, *n*)-mer and *k*-mer frequencies performance under different cases of sequence classification using ML methodologies. The (*m*, *n*)-mer algorithm is released as an R package within the CRAN project (https://cran.r-project.org/web/packages/mnmer) and is also available at https://github.com/labinfo-lncc/mnmer.

## 2 Methods

The methodology here presented and named (*m*, *n*)-mer is an extension of the *k*-mer concept. Let *S* be an observed nucleotide sequence. Let us denote by $s_{k}(n)$ the *k*-mer that starts at position *n* of this sequence. This string can only assume values in the set {AA..A,AA..C,…,TT…T} with $4^{k}$ distinct possibilities.

To ease our notation in future discussions, let us start considering the association of bases A, C, G and T with the digits 0, 1, 2 and 3, univocally but in any order. Hence, a base-4 integer number can characterize any *k*-mer. For example, the 6-mer ACCTGA can be characterized by the base-4 number 011320, corresponding to the decimal number 376. Hence, *k*-mers can now be associated with numerical values in the integer set ranging from 0 to $4^{k}-1$.

**Figure 1.**
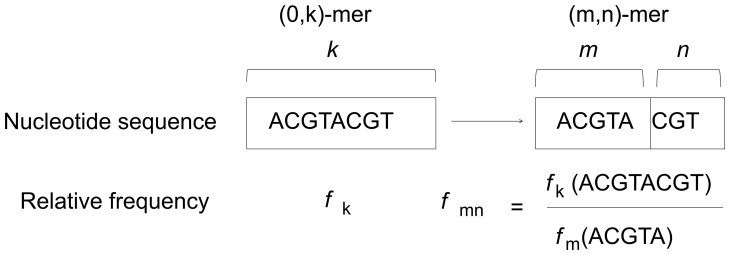
The relationship between *k*-mers and (*m*, *n*)-mers frequencies when *k* = *m* + *n*

Classification programs that use *k*-mers do employ as a feature a real-valued vector $4^{k}$ long where its *r*-th element is the probability of observing *k*-mer (r−1) in a sequence *S*, or more formally, this element is the $\mathrm{prob}\{s_{k}(u)=r-1\}$ for some valid *u*. The (*m*, *n*)-mer concept initially stems from considering the Markov chain as the mechanism that can statistically characterize nucleotide sequences (Ren *et al.*, 2018). Understanding *k*-mer as the state of this Markov chain, the corresponding probability transition matrix *P* of degree *d* has dimension $\left(4^{k},4^{k}\right)$, and its (*i*, *j*)-element is the conditional probability $\mathrm{prob}\{s_{k}(u+d)=j-1|s_{k}(u)=i-1\}$. Commonly it is assumed that this Markov chain is stationary, implying the transition probabilities do not depend on *u*, identical to the case of the *k*-mer distributions. The greatest issue with this approach is that features now are $4^{2k}$ long, which can be prohibitive even for moderate values of *k*. The aim of the (*m*, *n*)-mer model is to disregard the Markov chain approach in *stricto sensu* while preserving the probability transition matrix *P*. The idea is to produce a classification feature equivalent to *k*-mer. One way of doing this is defining a matrix $P^{*}$ of dimension $\left(4^{m},4^{n}\right)$, where m+n=k, d=m, where its (*i*, *j*)-element is the conditional probability $\mathrm{prob}\{s_{n}(u+m)=j-1|s_{m}(u)=i-1\}$. It is assumed that these probabilities are also stationary. Figure 1 illustrates the relationship between all these mers.

These conditional probabilities can be estimated by the *k*-mers counting process as well. Using the numerical equivalence between numbers and nucleotide sequences previously discussed, let $N_{k}$ be the total number of *k*-mers in the sequence *S* and let $c_{k}^{v}$ be the total number of *k*-mers in the sequence *S* associated with decimal number *v*.

The feature vector for the *k*-mer is the vector $f_{k}=(f_{k}^{0},f_{k}^{1},\ldots ,f_{k}^{K-1})$, where $K=4^{k}$ and $f_{k}^{v}=c_{k}^{v}/N_{k}$ for *v* from 0 to K−1. If $N_{k}$ is zero, then $f_{k}^{v}$ is also zero.

By using classical formulas involving conditional probabilities, it is possible to show that the feature vector for the (*m*, *n*)-mer frequencies is the vector of the same size as the above: $f_{m,n}=(f_{m,n}^{0,0},f_{m,n}^{0,1},\ldots ..,f_{m,n}^{0,N-1},\ldots ..,f_{m,n}^{M-1,0},f_{m,n}^{M-1,1},\ldots ..,f_{m,n}^{M-1,N-1})$, where $M=4^{m}$, $N=4^{n}$ and $f_{m,n}^{u,v}=f_{m+n}^{M.u+v}/f_{m}^{u}$ for *u* from 0 to M−1, and *v* from 0 to N−1. Notice that (*m*, *n*)-mer frequencies are calculated from *k*-mer frequencies for the two cases where k=m+n and k=m.

The elements of *k*-mer feature vector $f_{k}$ sum to 1 while the elements of (*m*, *n*)-mer feature vector $f_{m,n}$ sum to *M*. Hence, if we normalize this vector by dividing it by *M*, we have an indistinguishable vector from $f_{k}$ in any sense. Any software using *k*-mer frequencies can also function with (*m*, *n*)-mer frequencies without modification, regardless of its application.

### 2.1 Datasets and sequence preprocessing for performance comparison

We selected 11 distinct polyphyletic datasets with varying levels of complexity to compare the performance of (*m*, *n*)-mers versus *k*-mers models (Supplementary Material S1). The binary classification used datasets 1–3, while multiclass classification employed datasets 4–6. Dataset 1 contains sequences used by the V-Classifier software (Alam and Chowdhury, 2020), whereas dataset 2 has sequences used to build the HumanVirFinder software (Zhang *et al.*, 2019). In contrast, datasets 3–6 were hand-curated and comprised complete viral genomes retrieved from the National Center for Biotechnology Information’s RefSeq database (NCBI). Only 2018–2022 releases were retrieved to reduce data processing time. Subsequently, we removed sequences with non-ACTG bases exceeding 2% of the genome length. By curating all datasets at a 90% identity, redundancies were removed using CD-HIT-est software (http://weizhong-cluster.ucsd.edu/cd-hit/). Datasets 7–11 consisted of synthetic metagenomic contigs selected for clustering purposes. These datasets were generated using complex bacterial communities and comprised a diverse range of clusters, ranging from 25 (dataset 7) to 1074 (dataset 11). Distinct clustering algorithms have been previously applied to these datasets (Kang *et al.*, 2015; Lin and Liao, 2016). Supplementary Material S1 presents detailed information regarding all input data.

Datasets from 1 to 11 were processed to generate independent feature matrices by means of the (*m*, *n*)-mer R package. The *k*-mer and (*m*, *n*)-mer frequency features are, respectively, referenced as (0, *k*) and (*m*, *n*) when calling the mnmer function of this package. This is the main function that converts biological data into numerical values. It receives a FASTA file as input and generates a table describing the conditional frequency distribution of the sequence’s selected (*m*, *n*)-mer frequencies. This output is combined with class information to generate the feature matrix for classification. To test performance on different sequence lengths, the genomes from each dataset used for binary classification were independently fragmented into 10 000, 5000, 3000, 1000 and 300 bp sequences. Each genome has produced no more than three non-overlapping fragments. All fragment lengths were processed independently to generate feature matrices.

To constrain the computational cost, *k* has ranged from 2 to 5. We generated 4 *k*-mer and 10 (*m*, *n*)-mer frequency distribution matrices, where m+n=k for each fragment or genome obtained from the datasets. Alam and Chowdhury (2020) used dataset 1 in conjunction with a unique feature extraction method, which combines different *k*-mers frequencies into one matrix. We used exactly the same dataset and feature extraction method except for the substitution of *k*-mer by (*m*, *n*)-mer frequencies. This differs from the techniques utilized in our other analyzed datasets.

### 2.2 Classification using ML algorithms

The first six datasets were analyzed using random resamplings, cross-validation strategies and different ML algorithms. To achieve data balance and avoid possible overfitting, the classification was executed for 50 random resamplings, with 500 sequences per class for the train and test set. For each resampling, we trained the models using the train function of the caret package with automatic parameter tuning (topepo.github.io/caret/). To evaluate performance, we have used the 10-fold cross-validation repeated three times with random splits. The flowchart of this methodology can be visualized in Supplementary Figure S2.

We processed datasets 1 and 2 using software scripts previously published by Alam and Chowdhury (2020) and Zhang *et al.* (2019), respectively. We applied leave-one-out cross-validation in conjunction with logistic regression for the classification of dataset 1. For dataset 2, we utilized the *K*-nearest neighbor (KNN) algorithm. The only modification we made to the original scripts was replacing the feature matrix, changing it from *k*-mer to (*m*, *n*)-mer frequencies.

To analyze dataset 3, we used the ExtraTrees algorithm. Contrastingly, we classified datasets 4–6 using the following algorithms: KNN, Random Forest, C5 tree, semisupervised discriminant analysis and penalized discriminant analysis. These analyses were conducted using the Caret package with default parameters in R language.

### 2.3 Clustering using multiple algorithms

For datasets 7–11, we extracted features associated with 4-mer, (1,3)-mer, (2,2)-mer and (3,1)-mer. All feature matrices were used in the following clustering algorithms: Affinity Propagation, Agglomerative clustering, Bisecting KMeans, KMeans, Gaussian-Mixture, MiniBatchKMeans and Spectral. The Python 3 programming language was utilized to implement them using Scikit-learn (scikit-learn.org) to ensure consistency and reproducibility of results across datasets. The mean-adjusted Rand index (ARI) was used to compare feature performance in conjunction with three replicate runs for each algorithm. For reproducibility purposes, the input data, output metrics and customized scripts used in our analysis are available at github.com/labinfo-lncc/mnmer.

## 3 Results and discussion

Our study compared the performance of (*m*, *n*)-mers and *k*-mers frequencies across 11 polyphyletic datasets, which included three for binary, three for multiclass and five for clustering classifications. All datasets were selected to represent potential classification challenges. These datasets present sequences labeled solely based on evolutionary origin or host information, presenting varying levels of taxonomic complexity (Supplementary Material S1). Traditional alignment-based approaches may struggle to identify group assignments for these taxonomically diverse datasets (Ren *et al.*, 2018; Zielezinski *et al.*, 2019). Consequently, alignment-free methods offer an alternative approach for classifying these highly divergent sequences. In this context, we showed that (*m*, *n*)-mers conditional probabilities may be a more convenient feature for classifying highly divergent sequences, even when addressing multiple classification tasks.

**Figure 2.**
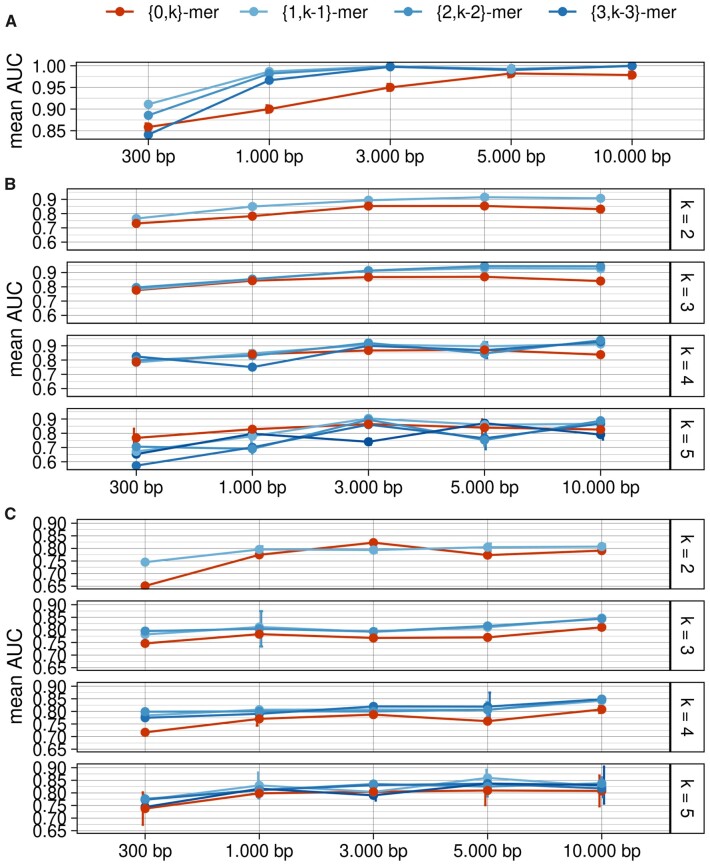
Classification performance of *k*-mer and (*m*, *n*)-mer on independent datasets after 50 random resamplings. This figure shows the mean and confidence interval. (**A**) Dataset 1 was first used by V-Classifier (Alam and Chowdhury, 2020). (**B**) Dataset 2, used by Hu-manVirFinder (Zhang *et al.*, 2019) and (**C**) Dataset 3, used to classify phages and viruses that do not infect bacteria

For binary classification, the (*m*, *n*)-mer performances have often surpassed those associated with corresponding *k*-mers, especially for smaller fragment sizes (Fig. 2 and Supplementary Table S1). The alignment-free classification of fragments ranging from 300 to 1000 bp is a well-known challenge due to its unspecific nature. Previously published *k*-mer-based computer programs were only effective for analyzing sequences larger than 1000 bp (Alam and Chowdhury, 2020; Ren *et al.*, 2018), which can limit results for shorter or fragmented sequences lacking complete predicted genes. The use of (*m*, *n*)-mers instead of *k*-mers may overcome these limitations in some datasets and allow accurate classification of sequences as short as 300 bp.

Furthermore, our binary classification analysis showed that in some cases, higher performance can be achieved using (*m*, *n*)-mers instead of *k*-mers in cases where *m* + *n* is less than *k*. For instance, when classifying 10 000 bp fragments in dataset 2, the (2,1)-mer (AUC = 0.9441) outperformed the (0,3)-mer (AUC = 0.8404), (0,4)-mer (AUC = 0.8377) and the (0,5)-mer (AUC = 0.8261) (Fig. 2 and Supplementary Table S1). Assuming that classification processing time is monotonically related to the *k*-value (Supplementary Material S2), in this example, better performance can be achieved 4–16 times faster using (*m*, *n*)-mer instead of *k*-mer. This can be highly relevant when dealing with biological big data. Additionally, the ternary hypothesis test of difference of means revealed that for dataset 2, except for the case *k* = 5, 300 bp and 1,000 bp, there was always an (*m*, *n*)-mer with performance superior to the corresponding *k*-mer. For dataset 3, the only exception occurred for *k* = 2 and 3000 bp (Supplementary Material S3).

In the multiclass classification, the (*m*, *n*)-mers demonstrate fairly superior performance metrics compared with corresponding *k*-mers when five ML algorithms are employed (Supplementary Fig. S3 and Supplementary Table S2). For datasets 4 and 6 and all classification algorithms, the (*m*, *n*)-mer performance always exceeded 0.95, while the *k*-mer performance never exceeded 0.90. For dataset 5, the (*m*, *n*) superiority is not so evident as in the two previous cases, but in all of them, the median performances of (*m*, *n*)-mers are higher than those of *k*-mers. These findings reinforce the advantages of using (*m*, *n*)-mers in biological sequence classification.

**Figure 3.**
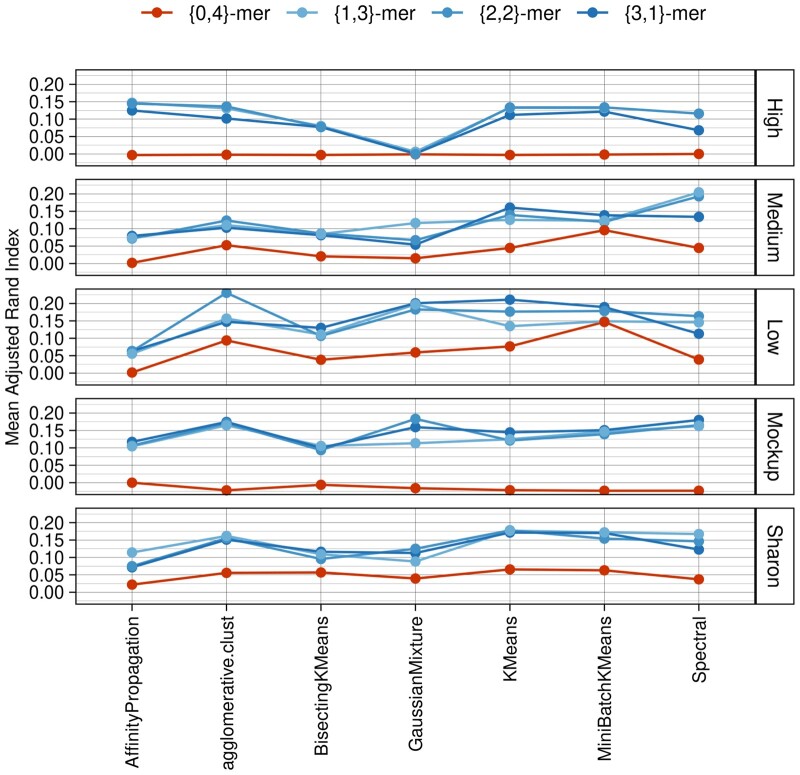
Clustering performance of (*m*, *n*)-mers and *k*-mers, when *k* = 4 using three replicates for five datasets

Similarly, the clustering of synthetic datasets 7–11 demonstrated that (*m*, *n*)-mers frequencies are a more suitable descriptor for intricate bacterial communities than *k*-mers (Fig. 3 and Supplementary Table S3). For the specific case of *k* = 4 and using ARI as a performance measure, a similar ternary hypothesis test has revealed that (*m*, *n*)-mers showed higher performance in all cases but one, in which a tie was observed (Supplementary Material S3). These results point out that (*m*, *n*)-mer frequencies could be used as a drop-in replacement to *k*-mers and improve the classification of small sequence fragments from complex polyphyletic datasets even for short values of *k*.

## 4 Conclusion

We propose a simple alternative alignment-free feature extraction method named (*m*, *n*)-mer, a variant of the traditional *k*-mer, that alternatively uses conditional probabilities. Our results revealed that it can accurately describe complex discriminatory patterns between biological sequences, even when using short values for *k* (ranging from 2 to 5) and smaller fragment lengths (from 300 to 1000 bp). This feature has often outperformed the *k*-mer in several cases for 11 distinct datasets, which addresses the tasks of binary, multiclass and clustering classifications. The (*m*, *n*)-mer frequencies can replace the *k*-mer frequencies in biological applications without any computational modification in computer programs involving the latter. This seems to be a quite considerable aspect from a practical standpoint. Consequently, performance should be the sole criterion by which one or the other should be preferred. We encourage researchers to apply the (*m*, *n*)-mer concept to their tasks and evaluate its adequacy to the case.

## Supplementary Material

vbad088_Supplementary_Data

## Data Availability

The data underlying this article are available at https://github.com/labinfo-lncc/mnmer in the article and in its online supplementary material.
