# Supplementary material for: (m, n)-mer—a simple statistical feature for sequence classification
Source: Bioinform Adv. 2023 Jul 11;3(1):vbad088. doi: 10.1093/bioadv/vbad088 (PMC10338135; doi:10.1093/bioadv/vbad088)
Supplement: vbad088_Supplementary_Data [file vbad088_supplementary_data.zip › Supplementary_Material_1. Andrade_et_al.pdf]

Supplementary Material 1. Detailed information on the datasets.

1. Data Manifest

The classification datasets (Table 1) present polyphyletic sequences where labeling is solely based on evolutionary origin (datasets one, four, and six) or host information (datasets two, three, and five). In contrast, all clustering datasets are synthetic metagenomic contigs generated by diverse microbial communities and present an increasing number of expected clusters.

Nonetheless, all raw data accession numbers, fragments, and scripts used in this project are stored at github.com/labinfo-lncc/mnmer. If you require access to any of the files not listed above, please open an issue on GitHub or email atrv@lncc.br

1.1 Classification datasets

Table 1. Summary of independent datasets used for binary and multiclass classification. The table shows the number of complete sequences for each class after removing redundancies.

Dataset	Class 1	Class 2	Class 3	Class 4
#1*	Viral RNA (n. 3,137)	Human RNA (n. 12,499)	-	-
#2**	Human Infecting Viruses (n. 1,210)	Other viruses (n. 8,116)	-	-
#3	Phage (n. 1,559)	Viruses that do not infect bacteria (n. 2,076)	-	-
#4	Amarillovirales (n. 7,166)	Bunyavirales (n. 1,764)	Mononegavirales (n. 4,802)	Nidovirales (n. 3,846)
#5	Arthropoda Infecting Viruses (11,236)	Phage (n. 1,559)	Mammalia Infecting Viruses (18,059)	Plant Infecting Viruses (28,581)
#6	Linear viral genomes (n. 7,000)	Bisegmented viral genomes (n. 2,122)	Trisegmented viral genomes (n. 1,764)	Multisegmented viral (n. 4,802)

*Curated and previously tested by Alam M. and Chowdhury, U. (2020).

** Curated and previously tested by Zhang, Z., *et al.* (2019).

1.2 Clustering datasets

The first dataset presents 25 clusters divided into 1,893 contigs generated from a mock community (Mockup dataset from Kang et al., 2015. Available at: http://portal.nersc.gov/dna/RD/Metagenome_RD/MetaBAT/Software/Mockup/). The second dataset has 32 clusters and 2,294 contigs from complex gut samples (Sharon et al., 2013.

Available at: http://ggkbase.berkeley.edu/carrol/). The third, fourth, and fifth datasets are from the CAMI challenge (Sczyrba et al., 2017. Available at: http://portal.nersc.gov/dna/RD/Metagenome_RD/MetaBAT/Files/CAMI/) with varying levels of complexity. The Low complexity dataset has 44 clusters and 19,499 contigs; the Medium complexity dataset includes 232 clusters and 63,447 contigs while the High complexity dataset contains 1,074 clusters and 42,038 contigs.

References

Alam M. and Chowdhury, U. (2020) Short K-Mer Abundance Profiles Yield Robust Machine Learning Features and Accurate Classifiers for RNA Viruses. PloS One, 15, e0239381.

Kang DD, Froula J, Egan R, Wang Z. 2015. MetaBAT, an efficient tool for accurately reconstructing single genomes from complex microbial communities. PeerJ 3:e1165

Sczyrba, A., Hofmann, P., Belmann, P. et al. Critical Assessment of Metagenome Interpretation—a benchmark of metagenomics software. Nat Methods 14, 1063–1071 (2017)

Sharon, I., Morowitz, MJ, Thomas, BC, Costello, EK, Relman, BA, Banfield, JF. Time series community genomics analysis reveals rapid shifts in bacterial species, strains, and phage during infant gut colonization. Genome Res. 2013. 23: 111-120

Lin, HH., Liao, YC. Accurate binning of metagenomic contigs via automated clustering sequences using information of genomic signatures and marker genes. Sci Rep 6, 24175 (2016)

Zhang, Z., *et al.* (2019) Rapid Identification of Human-Infecting Viruses. Transboundary and Emerging Diseases, 66, 2517–22.
