# Supplementary material for: (m, n)-mer—a simple statistical feature for sequence classification
Source: Bioinform Adv. 2023 Jul 11;3(1):vbad088. doi: 10.1093/bioadv/vbad088 (PMC10338135; doi:10.1093/bioadv/vbad088)
Supplement: vbad088_Supplementary_Data [file vbad088_supplementary_data.zip › Supplementary_Material_2. Andrade_et_al.pdf]

Supplementary Material 2. Processing time for (m,n)-mers and k-mers generation.

We performed feature matrix generation of 3-mer, 4-mer, and 5-mer, as well as for all corresponding (m,n)-mers (Figure 1). The mnmer R package was used and generated features for 100 replicates. It ran on a server with AMD EPYC 7313P 16-Core Processor, 128 Gb RAM, 1.2 TB of HD space, and SSD of 1.2 TB. Our server had 32 Threads, however, we only used 1 thread for this analysis. In all of our observations, the standard deviation was less than 0.021.

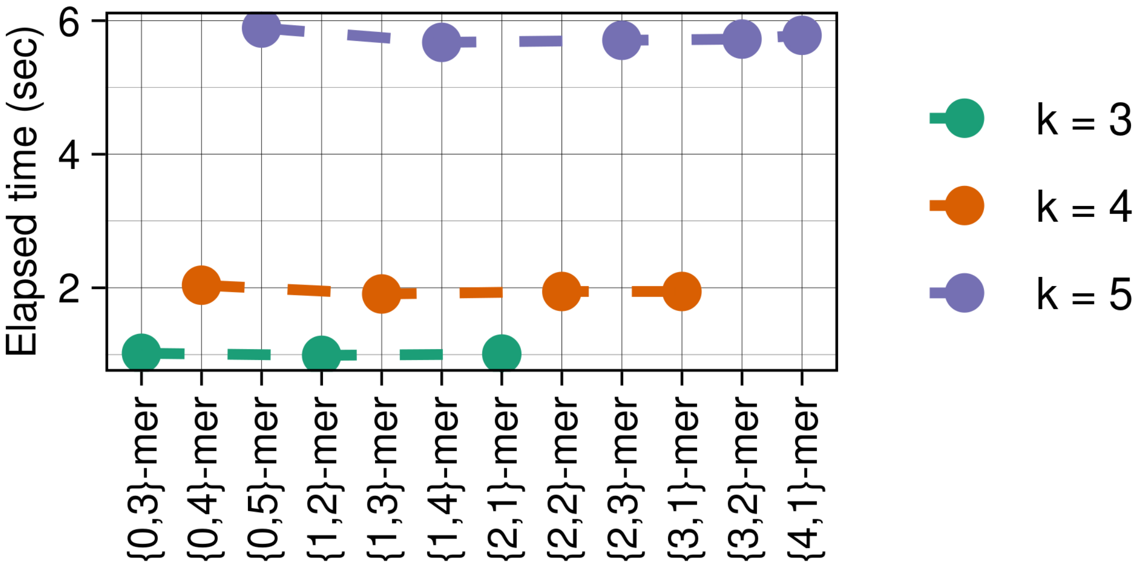

Figure 1. Comparing processing time for (m,n)-mers and k-mers generation.

The input data and scripts used for benchmarking and plotting are available at: https://github.com/labinfo-lncc/mnmer
