# Supplementary material for: (m, n)-mer—a simple statistical feature for sequence classification
Source: Bioinform Adv. 2023 Jul 11;3(1):vbad088. doi: 10.1093/bioadv/vbad088 (PMC10338135; doi:10.1093/bioadv/vbad088)
Supplement: vbad088_Supplementary_Data [file vbad088_supplementary_data.zip › Supplementary_Material_3.Andrade_et_al.pdf]

Supplementary Material 3. Hypothesis test results.

The comparison between (m,n)-mer e k-mers for datasets two and three were made by a ternary hypothesis test as follows:

Defining μ_{mn} and μ_k as respectively the performance expected value for the (m,n)-mer and the k-mer, this test is made on the hypotheses **H0 :**$\mu_{mn} < \mu_k$, **H1 :**$\mu_{mn} > \mu_k$ and **H2 :**$\mu_{mn} = \mu_k$, based on 500 observations of each case. The confidence level was 95%.

DATASET TWO

k	4^k	(m,n)	300 bp	1000 bp	3000 bp	5000 bp	10000 bp
2	16	(1,1)	H1	H1	H1	H1	H1
3	64	(1,2)	H1	H1	H1	H1	H1
		(2,1)	H1	H1	H1	H1	H1
4	256	(1,3)	H0	H1	H1	H1	H1
		(2,2)	H1	H0	H0	H0	H1
		(3,1)	H1	H0	H0	H0	H1
5	1024	(1,4)	**H0**	**H0**	H1	H1	H1
		(2,3)	**H0**	**H0**	H1	H0	H1
		(3,2)	**H0**	**H0**	H0	H0	H1
		(4,1)	**H0**	**H0**	H0	H1	H0

DATASET THREE

K	4^K	(m,n)	300 bp	1000 bp	3000 bp	5000 bp	10000 bp
2	16	(1,1)	H1	H1	H0	H1	H1
3	64	(1,2)	H1	H1	H1	H1	H1
		(2,1)	H1	H1	H1	H1	H1
4	256	(1,3)	H1	H1	H1	H1	H1
		(2,2)	H1	H1	H1	H1	H1
		(3,1)	H1	H1	H1	H1	H1
5	1024	(1,4)	H1	H1	H2	H1	H1
		(2,3)	H1	H1	H1	H1	H1
		(3,2)	H1	H1	H1	H1	H1
		(4,1)	H1	H1	H0	H1	H1

Except for the cases (k=5, 300 bp) and (k=5, 1000 b), for each value of k, there is an associated (m,n)-mer that outperforms the k-mer. One possible explanation for why this fact does not happen in the mentioned cases is because the number of bases in the sequence is smaller than the feature size. This will prevent the adequate evaluation of the feature's frequencies.

For datasets six to eleven (table below), the hypothesis testing was the same. It was performed for three replicates.

Clustering Algorithm	Sharon			Mockup			Low			Medium			High		
	(1,3)mer	(2,2)mer	(3,1)mer	(1,3)mer	(2,2)mer	(3,1)mer	(1,3)mer	(2,2)mer	(3,1)mer	(1,3)mer	(2,2)mer	(3,1)mer	(1,3)mer	(2,2)mer	(3,1)mer
Affinity Prop.	**H1**	**H1**	**H1**	**H1**	**H1**	**H1**	**H1**	**H1**	**H1**	**H1**	**H1**	**H1**	**H1**	**H1**	**H1**
Bisect. K-means	**H1**	**H1**	**H1**	**H1**	**H1**	**H1**	**H1**	**H1**	**H1**	**H1**	**H1**	**H1**	**H1**	**H1**	**H1**
Gaussian Mix.	**H1**	**H1**	**H1**	**H1**	**H1**	**H1**	**H1**	**H1**	**H1**	**H1**	**H1**	**H2**	**H2**	**H2**	**H2**
K-means	**H1**	**H1**	**H1**	**H1**	**H1**	**H1**	**H1**	**H1**	**H1**	**H1**	**H1**	**H1**	**H1**	**H1**	**H1**
MBatch K-means	**H1**	**H1**	**H1**	**H1**	**H1**	**H1**	**H1**	**H1**	**H1**	**H1**	**H1**	**H1**	**H1**	**H1**	**H1**
Spectral	**H1**	**H1**	**H1**	**H1**	**H1**	**H1**	**H1**	**H1**	**H1**	**H1**	**H1**	**H1**	**H1**	**H1**	**H1**
Agglomerative	**H1**	**H1**	**H1**	**H1**	**H1**	**H1**	**H1**	**H1**	**H1**	**H1**	**H1**	**H1**	**H1**	**H1**	**H1**
