# Supplementary material for: (m, n)-mer—a simple statistical feature for sequence classification
Source: Bioinform Adv. 2023 Jul 11;3(1):vbad088. doi: 10.1093/bioadv/vbad088 (PMC10338135; doi:10.1093/bioadv/vbad088)
Supplement: vbad088_Supplementary_Data [file vbad088_supplementary_data.zip › SupplementaryTable 1. Andrade_et_al.pdf]

Supplementary Table S1. Performance metrics for binary classification.

(m,n)-mers	Mean Metrics						Fragment
	Accuracy	Specificity	Sensitivity	Precision	F1-Score	AUC	
(0,k)-mer	8.784	9.501	8.784	8.695	8.718	8.585	Dataset1_300bp
(1,k-1)-mer	8.891	9.537	8.891	8.822	8.840	9.111	Dataset1_300bp
(2,k-2)-mer	8.843	9.591	8.843	8.752	8.763	8.855	Dataset1_300bp
(3,k-3)-mer	8.561	9.521	8.561	8.388	8.418	8.409	Dataset1_300bp
(0,k)-mer	9.298	9.308	9.419	9.280	9.288	9.000	Dataset1_1000bp
(1,k-1)-mer	9.565	8.378	9.565	9.558	9.560	9.867	Dataset1_1000bp
(2,k-2)-mer	9.499	9.774	9.499	9.488	9.491	9.816	Dataset1_1000bp
(3,k-3)-mer	9.444	9.766	9.444	9.430	9.432	966	Dataset1_1000bp
(0,k)-mer	9.771	9.675	9.743	9.741	9.769	9.500	Dataset1_3000bp
(1,k-1)-mer	9.876	9.929	9.876	9.876	9.876	9.990	Dataset1_3000bp
(2,k-2)-mer	9.835	9.915	9.835	9.834	9.834	9.984	Dataset1_3000bp
(3,k-3)-mer	9.820	9.895	9.820	9.812	9.819	9.975	Dataset1_3000bp
(0,k)-mer	9.563	9.779	9.563	9.566	9.561	9.822	Dataset1_5000bp
(1,k-1)-mer	9.602	9.823	9.602	9.606	9.600	9.932	Dataset1_5000bp
(2,k-2)-mer	9.600	9.784	9.600	9.606	9.599	992	Dataset1_5000bp
(3,k-3)-mer	9.837	9.905	9.837	98.374	9.837	9.902	Dataset1_5000bp
(0,k)-mer	9.915	9.966	9.899	9.951	9.919	9.786	Dataset1_10000bp
(1,k-1)-mer	9.981	9.974	9.971	9.971	9.970	9.998	Dataset1_10000bp
(2,k-2)-mer	9.991	9.984	9.971	9.916	9.917	9.999	Dataset1_10000bp
(3,k-3)-mer	9.957	9.990	9.957	9.957	9.957	9.996	Dataset1_10000bp
(0,2)-mer	7.163	7.424	7.163	8.338	7.575	7.311	Dataset2_300bp
(1,1)-mer	7.463	7.627	7.463	8.495	7.818	7.654	Dataset2_300bp
(0,3)-mer	7.313	7.535	7.313	8.376	7.690	7.764	Dataset2_300bp
(1,2)-mer	7.324	7.603	7.324	8.318	7.687	7.820	Dataset2_300bp
(2,1)-mer	7.378	7.646	7.378	8.350	7.731	7.952	Dataset2_300bp
(0,4)-mer	6.892	7.100	6.422	8.014	7.081	7.868	Dataset2_300bp

(1,3)-mer	6.992	7.159	6.922	8.214	7.381	7.837	Dataset2_300bp
(2,2)-mer	7.469	7.905	7.469	8.207	7.762	7.988	Dataset2_300bp
(3,1)-mer	7.227	7.677	7.227	8.099	7.574	8.246	Dataset2_300bp
(0,5)-mer	7.295	7.609	7.495	8.084	7.745	7.676	Dataset2_300bp
(1,4)-mer	7.694	8.336	7.694	8.084	7.867	6.723	Dataset2_300bp
(2,3)-mer	8.279	8.710	7.517	8.083	7.126	7.063	Dataset2_300bp
(3,2)-mer	7.672	8.434	7.672	7.945	7.800	6.731	Dataset2_300bp
(4,1)-mer	7.924	8.619	7.924	8.132	8.021	6.538	Dataset2_300bp
(0,2)-mer	8.498	8.656	8.498	8.921	8.643	7.823	Dataset2_1000bp
(1,1)-mer	7.828	7.868	7.828	8.766	8.127	8.496	Dataset2_1000bp
(0,3)-mer	8.058	8.182	8.058	8.767	8.296	8.430	Dataset2_1000bp
(1,2)-mer	8.107	8.268	8.107	8.748	8.327	9.304	Dataset2_1000bp
(2,1)-mer	8.150	8.324	8.150	8.753	8.359	9.456	Dataset2_1000bp
(0,4)-mer	8.000	8.188	8.018	8.676	8.236	8.404	Dataset2_1000bp
(1,3)-mer	8.254	8.374	8.578	8.886	8.621	8.963	Dataset2_1000bp
(2,2)-mer	8.078	8.225	8.012	8.639	8.228	8.461	Dataset2_1000bp
(3,1)-mer	7.980	8.200	7.980	8.449	8.207	8.688	Dataset2_1000bp
(0,5)-mer	7.764	7.523	7.764	8.365	8.109	8.283	Dataset2_1000bp
(1,4)-mer	7.780	8.410	7.780	8.134	7.937	8.594	Dataset2_1000bp
(2,3)-mer	7.394	7.670	7.394	8.347	7.742	7.521	Dataset2_1000bp
(3,2)-mer	7.860	8.453	7.860	8.013	8.208	7.649	Dataset2_1000bp
(4,1)-mer	7.957	8.595	7.957	8.069	8.206	8.703	Dataset2_1000bp
(0,2)-mer	8.386	8.176	8.380	8.929	8.609	8.528	Dataset2_3000bp
(1,1)-mer	8.380	8.434	8.380	8.969	8.567	8.937	Dataset2_3000bp
(0,3)-mer	8.498	8.496	8.498	9.075	8.672	8.679	Dataset2_3000bp
(1,2)-mer	8.321	8.416	8.321	8.904	8.512	9.125	Dataset2_3000bp
(2,1)-mer	8.327	8.317	8.327	8.534	9.011	9.132	Dataset2_3000bp
(0,4)-mer	8.021	8.145	8.241	8.245	8.333	8.671	Dataset2_3000bp
(1,3)-mer	8.343	8.373	8.343	8.977	8.541	9.075	Dataset2_3000bp

(2,2)-mer	8.337	8.311	8.337	9.033	8.546	9.197	Dataset2_3000bp
(3,1)-mer	8.305	8.336	8.305	8.962	8.510	8.996	Dataset2_3000bp
(0,5)-mer	8.039	8.216	8.139	8.171	8.228	8.644	Dataset2_3000bp
(1,4)-mer	8.284	8.391	8.284	8.875	8.479	9.038	Dataset2_3000bp
(2,3)-mer	8.273	8.342	8.273	8.910	8.478	8.950	Dataset2_3000bp
(3,2)-mer	8.193	8.422	8.193	8.716	8.381	8.606	Dataset2_3000bp
(4,1)-mer	8.482	9.285	8.482	8.343	8.404	7.402	Dataset2_3000bp
(0,2)-mer	8.621	8.453	8.621	8.493	8.797	8.534	Dataset2_5000bp
(1,1)-mer	8.395	8.552	8.299	8.876	8.574	9.147	Dataset2_5000bp
(0,3)-mer	8.707	8.552	8.207	8.466	8.715	8.702	Dataset2_5000bp
(1,2)-mer	8.183	8.582	8.223	8.676	8.381	8.541	Dataset2_5000bp
(2,1)-mer	8.123	8.399	8.255	8.822	8.387	8.514	Dataset2_5000bp
(0,4)-mer	8.411	8.422	8.311	8.309	8.790	8.711	Dataset2_5000bp
(1,3)-mer	8.292	8.352	8.255	8.785	8.355	8.466	Dataset2_5000bp
(2,2)-mer	8.200	8.125	8.122	8.242	8.236	8.301	Dataset2_5000bp
(3,1)-mer	7.233	7.425	7.058	8.285	7.575	7.503	Dataset2_5000bp
(0,5)-mer	8.095	8.164	8.595	8.144	8.256	8.395	Dataset2_5000bp
(1,4)-mer	7.532	7.824	7.263	8.568	7.885	7.795	Dataset2_5000bp
(2,3)-mer	6.822	7.035	7.078	8.097	6.974	6.904	Dataset2_5000bp
(3,2)-mer	7.263	7.124	7.274	8.245	7.255	7.014	Dataset2_5000bp
(4,1)-mer	7.374	7.574	7.223	8.428	7.675	7.964	Dataset2_5000bp
(0,2)-mer	7.830	7.820	7.830	8.813	8.132	8.313	Dataset2_10000bp
(1,1)-mer	8.756	8.761	8.656	9.081	8.883	9.071	Dataset2_10000bp
(0,3)-mer	8.063	8.223	8.063	8.725	8.290	8.404	Dataset2_10000bp
(1,2)-mer	8.643	8.638	8.643	9.040	8.791	9.268	Dataset2_10000bp
(2,1)-mer	8.761	8.724	8.761	9.222	8.894	9.441	Dataset2_10000bp
(0,4)-mer	8.254	8.412	8.254	8.807	8.443	8.377	Dataset2_10000bp
(1,3)-mer	8.552	8.552	8.552	9.096	8.516	9.125	Dataset2_10000bp
(2,2)-mer	8.675	8.607	8.675	9.015	8.827	9.387	Dataset2_10000bp

(3,1)-mer	8.659	8.576	8.659	9.222	8.816	9.281	Dataset2_10000bp
(0,5)-mer	8.344	8.534	8.344	8.816	8.510	8.261	Dataset2_10000bp
(1,4)-mer	8.611	8.681	8.611	9.055	8.752	8.669	Dataset2_10000bp
(2,3)-mer	8.643	8.730	8.643	9.054	8.776	8.877	Dataset2_10000bp
(3,2)-mer	8.482	8.601	8.482	8.951	8.638	8.677	Dataset2_10000bp
(4,1)-mer	7.707	8.761	8.107	8.263	8.179	7.908	Dataset2_10000bp
(0,2)-mer	6.734	6.831	6.274	6.831	6.731	6.511	Dataset3_300bp
(1,1)-mer	7.263	7.224	7.163	8.238	7.675	7.454	Dataset3_300bp
(0,3)-mer	7.257	7.581	6.960	7.581	7.190	7.464	Dataset3_300bp
(1,2)-mer	7.755	8.255	7.552	8.355	8.133	7.820	Dataset3_300bp
(2,1)-mer	7.812	8.661	8.104	8.373	8.279	7.952	Dataset3_300bp
(0,4)-mer	7.210	7.210	6.108	6.600	6.620	7.168	Dataset3_300bp
(1,3)-mer	7.374	7.687	7.299	8.189	7.632	7.837	Dataset3_300bp
(2,2)-mer	7.755	8.554	7.772	8.277	8.231	7.988	Dataset3_300bp
(3,1)-mer	7.144	7.655	7.355	8.271	7.599	7.746	Dataset3_300bp
(0,5)-mer	7.000	7.131	7.031	7.044	6.987	7.376	Dataset3_300bp
(1,4)-mer	7.241	7.604	7.388	8.281	7.597	7.723	Dataset3_300bp
(2,3)-mer	7.274	7.647	7.355	8.305	7.611	7.763	Dataset3_300bp
(3,2)-mer	7.341	7.611	7.300	8.289	7.632	7.731	Dataset3_300bp
(4,1)-mer	7.199	7.425	7.158	8.085	7.475	7.438	Dataset3_300bp
(0,2)-mer	7.199	7.674	7.358	8.289	7.687	7.745	Dataset3_1000bp
(1,1)-mer	7.478	7.546	7.278	8.357	7.589	7.958	Dataset3_1000bp
(0,3)-mer	7.258	7.444	7.257	8.077	7.731	7.830	Dataset3_1000bp
(1,2)-mer	8.244	8.437	8.254	8.699	8.500	8.114	Dataset3_1000bp
(2,1)-mer	8.078	8.246	7.678	8.425	7.960	8.041	Dataset3_1000bp
(0,4)-mer	7.227	7.678	7.396	8.258	7.688	7.704	Dataset3_1000bp
(1,3)-mer	7.578	8.146	7.378	8.450	7.931	8.066	Dataset3_1000bp
(2,2)-mer	7.400	7.688	7.374	8.357	7.775	8.001	Dataset3_1000bp
(3,1)-mer	7.247	7.536	7.377	8.158	7.689	7.903	Dataset3_1000bp

(0,5)-mer	7.358	7.758	7.458	8.458	7.691	7.983	Dataset3_1000bp
(1,4)-mer	8.111	8.325	8.511	8.573	8.687	8.295	Dataset3_1000bp
(2,3)-mer	8.144	8.574	8.374	8.789	8.577	8.104	Dataset3_1000bp
(3,2)-mer	8.212	8.544	8.312	8.800	8.499	8.114	Dataset3_1000bp
(4,1)-mer	8.248	8.541	8.399	8.841	8.489	8.164	Dataset3_1000bp
(0,2)-mer	8.021	8.253	7.821	8.393	8.344	8.228	Dataset3_3000bp
(1,1)-mer	7.374	7.674	7.375	8.355	7.785	7.937	Dataset3_3000bp
(0,3)-mer	7.299	7.589	7.374	8.385	7.731	7.679	Dataset3_3000bp
(1,2)-mer	7.374	7.589	7.747	8.585	7.569	7.925	Dataset3_3000bp
(2,1)-mer	7.199	7.589	7.485	8.369	7.785	7.932	Dataset3_3000bp
(0,4)-mer	7.148	7.500	7.358	8.328	7.782	7.871	Dataset3_3000bp
(1,3)-mer	7.878	8.246	8.078	8.850	8.021	8.075	Dataset3_3000bp
(2,2)-mer	7.578	8.246	7.578	8.450	7.831	7.997	Dataset3_3000bp
(3,1)-mer	8.284	8.489	8.599	8.885	8.585	8.196	Dataset3_3000bp
(0,5)-mer	7.800	8.105	7.377	8.385	7.712	8.044	Dataset3_3000bp
(1,4)-mer	7.796	8.156	7.855	8.385	7.969	8.038	Dataset3_3000bp
(2,3)-mer	8.161	9.256	8.325	8.358	8.258	8.350	Dataset3_3000bp
(3,2)-mer	8.011	8.341	8.322	8.394	8.441	8.306	Dataset3_3000bp
(4,1)-mer	7.374	7.674	7.385	8.345	7.712	7.902	Dataset3_3000bp
(0,2)-mer	7.254	7.553	7.224	8.289	7.687	7.734	Dataset3_5000bp
(1,1)-mer	7.574	7.746	7.568	8.450	7.931	8.047	Dataset3_5000bp
(0,3)-mer	7.324	7.603	7.324	8.318	7.687	7.702	Dataset3_5000bp
(1,2)-mer	7.978	8.246	7.978	8.655	7.963	8.104	Dataset3_5000bp
(2,1)-mer	7.998	8.300	7.965	8.458	7.964	8.156	Dataset3_5000bp
(0,4)-mer	7.174	7.788	7.524	8.358	7.654	7.611	Dataset3_5000bp
(1,3)-mer	7.586	7.674	7.386	8.385	7.796	8.063	Dataset3_5000bp
(2,2)-mer	7.855	8.236	7.589	8.758	7.858	8.061	Dataset3_5000bp
(3,1)-mer	8.378	8.596	8.333	8.793	8.486	8.188	Dataset3_5000bp
(0,5)-mer	7.966	8.236	7.963	8.369	7.966	8.095	Dataset3_5000bp

(1,4)-mer	8.544	9.444	8.258	8.247	8.365	8.594	Dataset3_5000bp
(2,3)-mer	8.274	8.855	8.383	8.758	8.485	8.252	Dataset3_5000bp
(3,2)-mer	8.141	9.021	8.261	8.148	8.418	8.364	Dataset3_5000bp
(4,1)-mer	8.041	8.473	8.121	8.033	8.577	8.370	Dataset3_5000bp
(0,2)-mer	7.478	7.746	7.477	8.299	7.831	7.913	Dataset3_10000bp
(1,1)-mer	7.758	8.005	7.585	8.369	7.966	8.471	Dataset3_10000bp
(0,3)-mer	8.054	8.364	8.028	8.589	8.282	8.104	Dataset3_10000bp
(1,2)-mer	8.161	9.221	8.261	8.411	8.358	8.468	Dataset3_10000bp
(2,1)-mer	8.241	9.141	8.544	8.288	8.418	8.441	Dataset3_10000bp
(0,4)-mer	7.578	8.100	7.962	8.850	7.931	8.077	Dataset3_10000bp
(1,3)-mer	8.271	9.555	8.544	8.312	8.422	8.425	Dataset3_10000bp
(2,2)-mer	8.461	9.500	8.571	8.348	8.399	8.487	Dataset3_10000bp
(3,1)-mer	8.474	9.671	8.471	8.378	8.388	8.481	Dataset3_10000bp
(0,5)-mer	7.878	8.246	7.878	8.950	7.931	8.017	Dataset3_10000bp
(1,4)-mer	8.358	8.599	8.347	8.785	8.458	8.269	Dataset3_10000bp
(2,3)-mer	8.296	8.589	8.236	8.785	8.510	8.377	Dataset3_10000bp
(3,2)-mer	8.044	8.234	8.044	8.516	8.344	8.177	Dataset3_10000bp
(4,1)-mer	8.141	9.371	8.211	8.048	8.118	8.308	Dataset3_10000bp
