# Supplementary figures and images for: (m, n)-mer—a simple statistical feature for sequence classification
Source: Bioinform Adv. 2023 Jul 11;3(1):vbad088. doi: 10.1093/bioadv/vbad088 (PMC10338135; doi:10.1093/bioadv/vbad088)

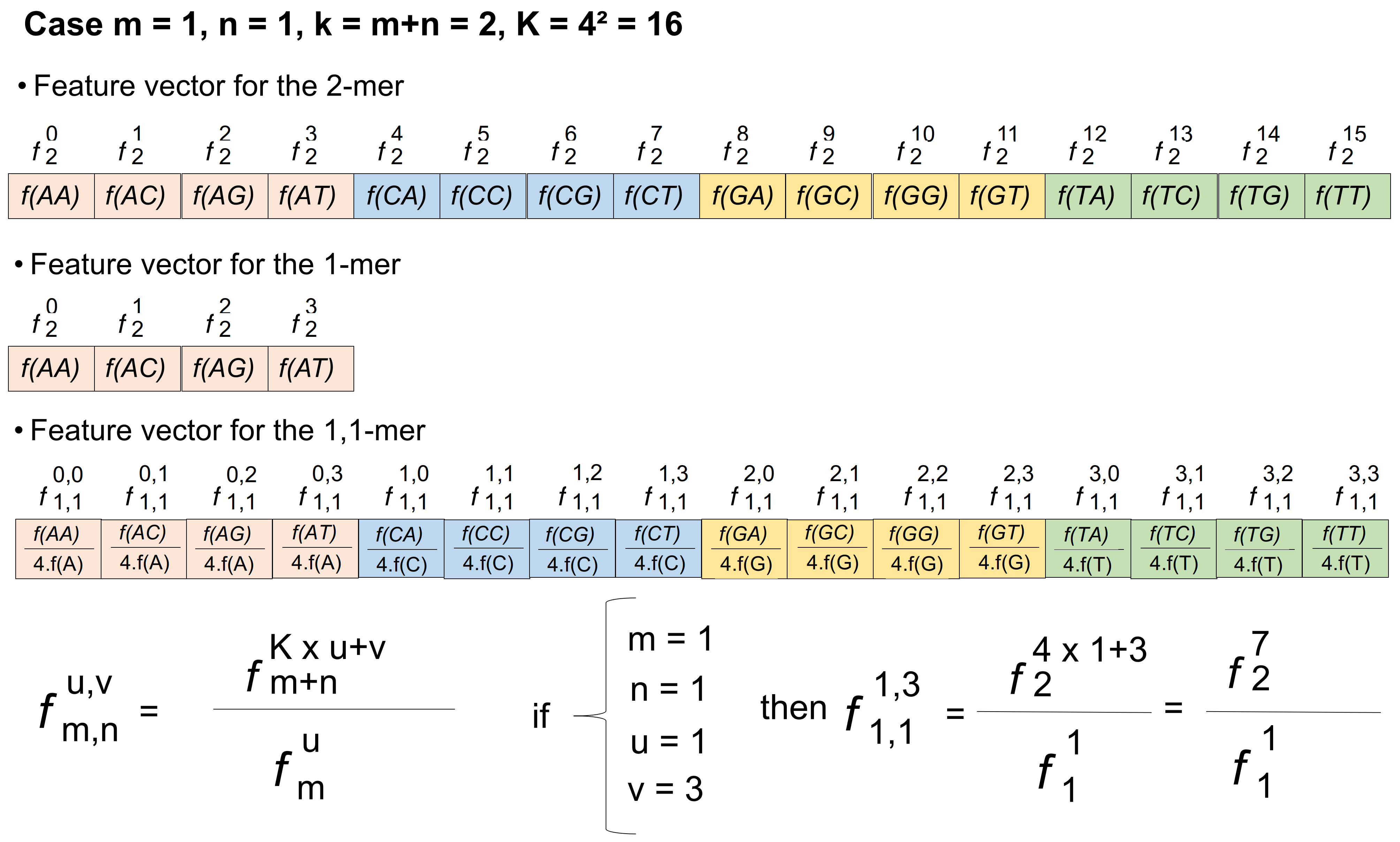

Supplement: vbad088_Supplementary_Data [file vbad088_supplementary_data.zip › SupplementaryFig1_Andrade_et_al.png]

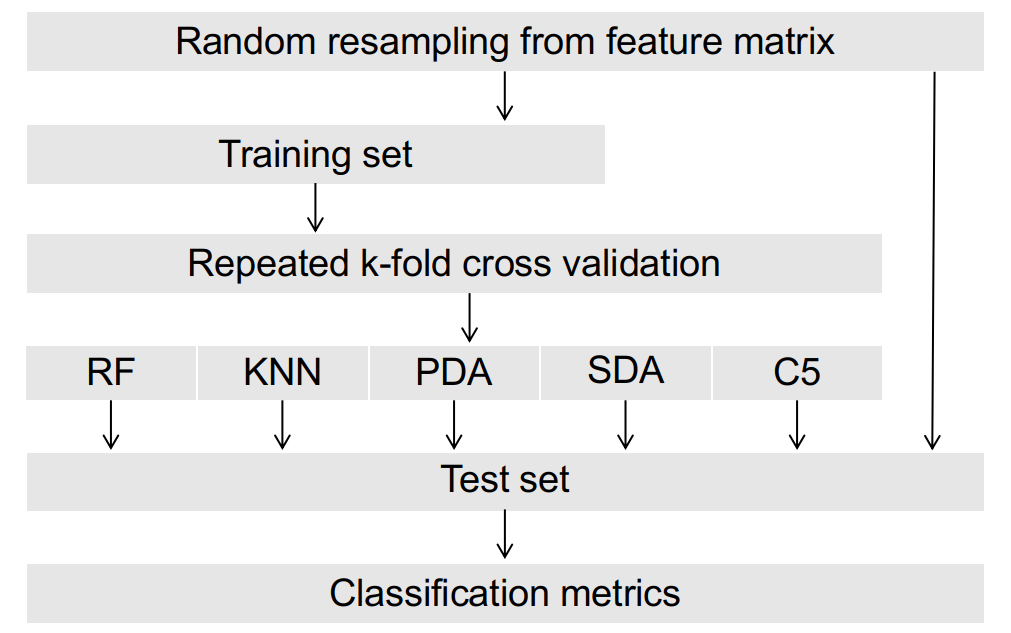

Supplement: vbad088_Supplementary_Data [file vbad088_supplementary_data.zip › SupplementaryFig2_Andrade_et_al.png]

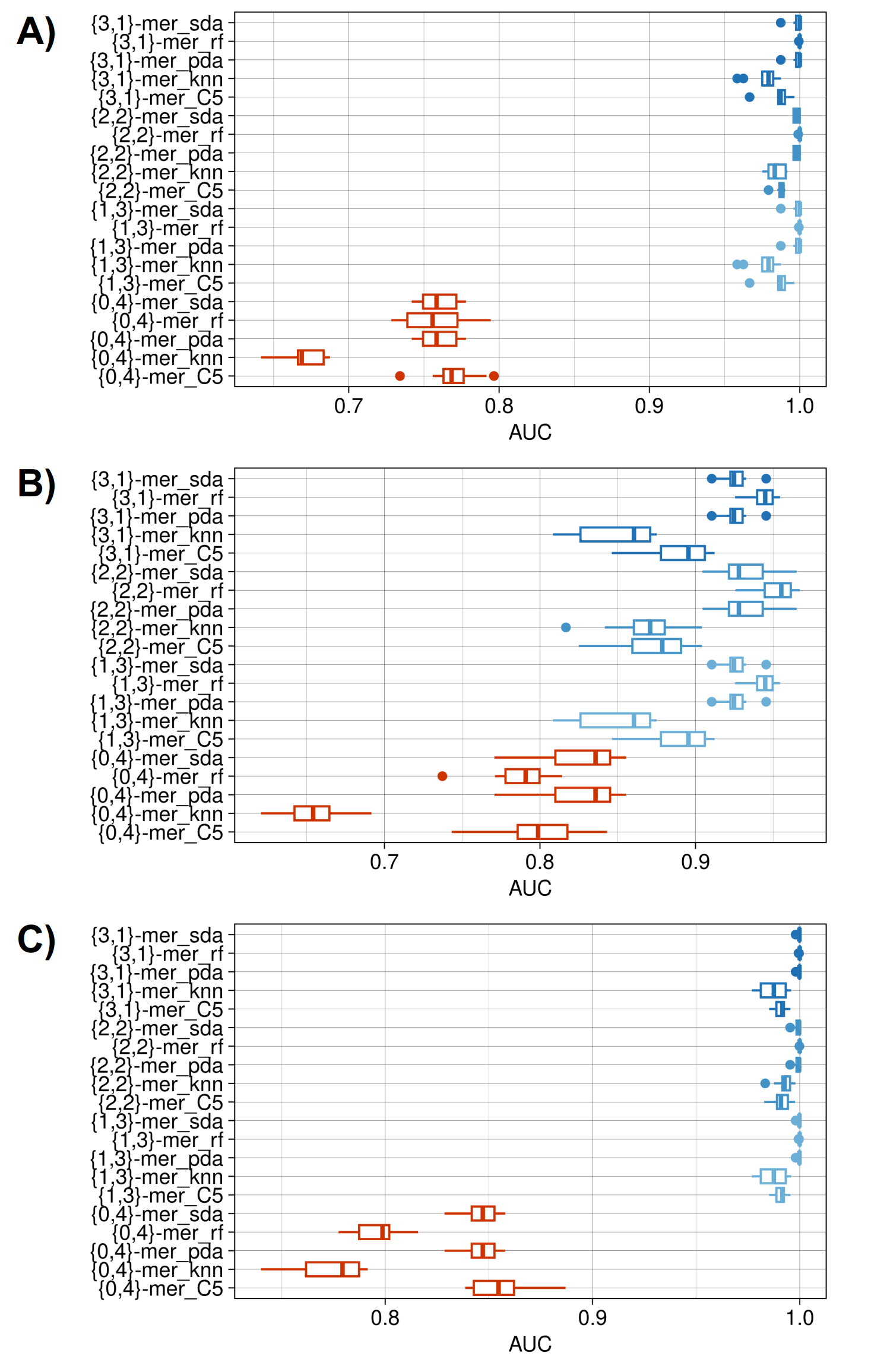

Supplement: vbad088_Supplementary_Data [file vbad088_supplementary_data.zip › SupplementaryFig3_Andrade_et_al.png]
